# Hemispheric Asymmetry in the Auditory Facilitation Effect in Dual-Stream Rapid Serial Visual Presentation Tasks

**DOI:** 10.1371/journal.pone.0104131

**Published:** 2014-08-13

**Authors:** Yasuhiro Takeshima, Jiro Gyoba

**Affiliations:** 1 Department of Psychology, Graduate School of Arts and Letters, Tohoku University, Sendai, Miyagi, Japan; 2 Department of Psychology, Graduate School of Arts and Letters, Tohoku University, Sendai, Miyagi, Japan; University of Tokyo, Japan

## Abstract

Even though auditory stimuli do not directly convey information related to visual stimuli, they often improve visual detection and identification performance. Auditory stimuli often alter visual perception depending on the reliability of the sensory input, with visual and auditory information reciprocally compensating for ambiguity in the other sensory domain. Perceptual processing is characterized by hemispheric asymmetry. While the left hemisphere is more involved in linguistic processing, the right hemisphere dominates spatial processing. In this context, we hypothesized that an auditory facilitation effect in the right visual field for the target identification task, and a similar effect would be observed in the left visual field for the target localization task. In the present study, we conducted target identification and localization tasks using a dual-stream rapid serial visual presentation. When two targets are embedded in a rapid serial visual presentation stream, the target detection or discrimination performance for the second target is generally lower than for the first target; this deficit is well known as attentional blink. Our results indicate that auditory stimuli improved target identification performance for the second target within the stream when visual stimuli were presented in the right, but not the left visual field. In contrast, auditory stimuli improved second target localization performance when visual stimuli were presented in the left visual field. An auditory facilitation effect was observed in perceptual processing, depending on the hemispheric specialization. Our results demonstrate a dissociation between the lateral visual hemifield in which a stimulus is projected and the kind of visual judgment that may benefit from the presentation of an auditory cue.

## Introduction

Multisensory integration aids the perception of the outer environment. If a particular sensory percept has low reliability, information from the other modality may, under certain conditions, compensate for such ambiguity. Many studies have examined the relationship between visual and auditory sensations [1]. For example, research has identified several phenomena, decreasing ambiguity of visual stimuli [2], increasing saliency of visual stimuli [3], and task performance being improved by sound [4]–[7].

According to the maximum likelihood estimation model, audio-visual integration is induced by utilizing optimal visual and auditory information [8]. In audio-visual integration, both, visual and auditory information compensate for the perceptual ambiguity associated with the other sensory modality [9], [10]. Generally, vision affords poorer temporal resolution than does the auditory modality [11]. Therefore, it is purported that auditory information compensates for poor visual temporal resolution [12]. Consistent with this proposal, sounds presented in synchrony with the onset of visual targets presented within rapid serial visual presentation (RSVP) stream improve target identification performance. For this type of presentation, two targets are embedded in the RSVP stream. In general, second target (T2) identification or detection performance is lower than that of the first target (T1); this deficit is well known as the attentional blink (AB) [13]. Moreover, participants often fail to detect repetitions of words in the RSVP stream; this phenomenon is known as repetition blindness (RB) [14]. Simultaneous presentation of a sound with the second target improves T2 identification or detection performance (decreasing the T2 deficit associated with the AB) [15]. In addition, synchronous sounds with two critical target characters facilitate T2 identification (thus avoiding failure to detect repetition in the RSVP stream due to RB) [16], [17]. Sounds which onset synchronize with the onset of visual targets in the RSVP stream aids in capturing the visual item and thus helps to segregate it from the RSVP stream [17].

Lateral (left/right) visual field anisotropies have been observed for a range of tasks. These anisotropies have been assumed to result from cerebral hemispheric asymmetries in the functioning of attentional mechanisms [18], [19]. For example, differences in attentional control between the cerebral hemispheres have been reported in studies on hemi-field neglect [20]; the left hemisphere (LH) has been found to control attention only in the right visual field (RVF) whereas the right hemisphere (RH) has been found to control attention in both the RVF and the left visual field (LVF). Additionally, the LVF is affected by contingent attentional capture [21]. Du and Abrams [21] suggested that the neural network mediating contingent attentional capture may be more lateralized in the RH than in the LH. Several fMRI studies have identified brain regions related to mediating contingent attentional capture; these include the intraparietal sulcus, the frontal eye fields, and the temporo-parietal junction [22], [23]. Further, Serences *et al*. [22] reported greater activation in the temporo-parietal junction on the right side than on the left side (although these hemispheric differences were not statistically significant).

In addition to differences in attentional function, hemispheric differences have also been observed for perceptual processing. The RVF-LH has been found to have advantage for verbal and linguistic processing (including letter identification) whereas the LVF-RH has been found to dominate in spatial processing [24]–[27]. In addition, for vision and audition left temporal areas are more specialized for temporal processing compared with right temporal areas [28], [29]. Indeed, the RVF-LH is involved in temporal processing [30] and is especially efficient for transient detection [31]. Auditory stimulus presentations would increase the RVF-LH activation because sound synchronous with a target itself becomes a cue for the temporal location of that target and transient detection for target is easier. Nakayama and Mackeben [32] have suggested that transient visual attention would be apparent at the primary visual cortex (V1). It is also known that auditory stimuli affect early visual processing [33], [34]. Therefore, the auditory facilitation effect should be more apparent in the RVF than in the LVF during the dual-stream RSVP task.

For the present study, we assumed another expectation of visual field asymmetry in the dual-stream RSVP task. Importantly attention to one sensory modality can spread to encompass simultaneous signals from another modality even when these other signals are task-irrelevant and from a different location [35]. In this case, cross-modal attentional spread combines attended visual input with an additional auditory stimulus, resulting in enhanced processing. We assume that attention is attracted toward the visual hemi-field according to hemispheric specialization. Therefore, auditory input may facilitate visual processing at the visual field corresponding to the cerebral hemisphere specialized for that visual processing.

We conducted the dual-stream RSVP task (i.e., the AB paradigm) to examine visual field asymmetry in auditory facilitation for visual processing. Previously, an AB deficit has been explained by a bottleneck model [36], [37] consisting of two sequential processing stages. In the first stage, processing is parallel and rapid whereas in the second stage processing is serial and slow. In the first stage, T1 and T2 receive sensory and perceptual encoding together. However, in the second stage, T2 cannot be processed during processing of T1. Therefore, T2 representation of T2 is reduced and may be subject to interruption by distracters. The previous research has shown that a synchronous sound makes visual object representation more robust [3]. Further, it is difficult to interrupt this audio-visual object representation using distracters. Presumably, attention is necessary for making these robust audio-visual object representations. Thus, in the dual-stream RSVP task, an auditory facilitation effect should be observed in the visual hemi-field (due to cross-modal attentional spread) according to hemispheric specialization.

On the other hand, we also predicted that the visual field asymmetry in auditory facilitation of visual processing did not depend on the lag condition. An AB deficit is generally observed with a small number of lags [13], thus it is assumed that the auditory facilitation effect should also be observed under these circumstances. However, synchronous sound produces robust object representation [3] and increased visual saliency [7]. This facilitation for visual presentation by simultaneous sound is also observed in backward masking paradigms [3]–[5]. Therefore, audio-visual object representation is robust and T2 identification performance would be improved regardless of temporal location between T1 and T2.

Many studies have examined visual field asymmetry in dual-stream RSVP tasks [38]–[41]. In these studies, participants identified two targets (T1 and T2) embedded in two simultaneously presented RSVP streams (i.e., the AB paradigm). Results indicate a clear LVF advantage as compared with the RVF even though the LVF-RH is associated with poorer temporal processing compared to the RVF-LH. Hollander *et al*. [38] concluded that an AB deficit is, unexpectedly, more related to spatial than temporal processing. Furthermore, ERP evidence suggests that this LVF advantage is due to an RH processing advantage, in which faster processing of distracters occurs in the RH from the onset of the trial; attentional selection of T2 proceeds faster when the target is presented on the left, and decision processes are better timed with T2 [42]. Moreover, LVF-RH processing has been shown to be efficient in sustained monitoring [31]. In the AB paradigm, sustained monitoring of the RSVP stream is required for detection of the two targets. Therefore, for the AB deficit, this hemispheric specialization may also be attributed to visual field asymmetry.

In the present study, we investigated whether a visual field asymmetry was present for the audio-visual synchrony effect on target identification during the RSVP paradigm. In Experiment 1, we used the target identification task to confirm the visual field asymmetry of the auditory facilitation effect. In Experiment 2, we re-examined the visual field asymmetry of the auditory facilitation effect by using a target identification task and by manipulating the experimental condition. We then conducted a target localization task to confirm the effect of the hemispheric specialization in Experiment 3.

## Experiment 1A

Using a dual-stream RSVP task, we compared the effects of an accompanying tone on the identification performance of T2 in the LVF and the RVF. In general, T2 performance is significantly poorer than T1 performance, but this deficit disappears with increasing temporal distance between T1 and T2 [13]. In addition, synchronous sound paired with T2 improves T2 performance [15]. We investigated the relationship between the facilitating effect of the audio-visual integration and the hemispheric asymmetry in temporal and target identification processing.

### Ethics statement

All experiments were approved by the ethics committee of the Graduate School of Arts and Letters, Tohoku University. All participants gave written informed consent prior to their participation.

### Participants

A group of nine, right-handed individuals (six women and three men) participated in Experiment 1A. They reported normal or corrected-to-normal vision and audition. Handedness was assessed with the Edinburgh Handedness Inventory [43]. In this test, a positive laterality quotient (LQ) score indicates that the participant is right-handed, while a negative LQ is indicative of left-handedness; those with LQ scores of 0 were considered mixed-handed. Mean LQ = 92 (*SD* = 12).

### Apparatus

The experimental stimuli were generated and controlled by means of a custom-made program written using MATLAB (MathWorks, Inc.), the Cogent Graphics and 2000 toolboxes (www.vislab.ucl.ac.uk/cogent.php), and a PC (Dell: XPS720). The visual stimuli were displayed on a CRT-display (SONY: Trinitron GDM-F520; resolution: 1024×768 pixels; refresh rate: 60 Hz). The auditory stimuli were conveyed through an audio interface (Roland: Edirol FA-66) and headphones (Sennheiser: HDA200). The simultaneity of the first or second targets and auditory stimuli was confirmed using a digital oscilloscope (Iwatsu: TS-80600). The experiment was conducted in a dark room with 43.6 dB (A) of background noise. The participants, their heads stabilized with a chin rest, viewed the monitor binocularly from a distance of 60 cm.

### Stimuli

**Figure 1:**
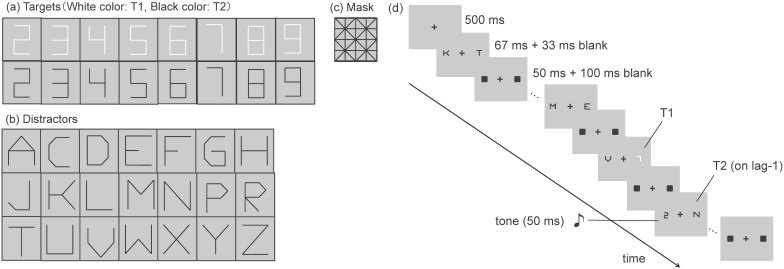
The visual stimuli and general paradigm used in the experiments. (a) Targets: Digits composed of line segments. White-colored digits were presented as T1, and black-colored digits were presented as T2. (b) Distracters: Letters composed of line segments (see Olivers & Van der Burg, 2008). (c) Mask stimulus: Grid pattern made from the same line segments used to make the targets and the distracters. (d) Outline of the general paradigm. T1 and T2 were presented randomly in the left (for half of the trials) or the right RSVP streams. T2 was presented at lag-1, lag-2 or lag-5 in Experiment 1A, and at lag-1, lag-3, or lag-5 in Experiment 1B. A tone was presented simultaneously with the onset of T2 in Experiments 1A, 2, and 3, and with either the onset of T1 or T2 in Experiment 1B. (With the permission from Japanese Psychonomic Society (JPS), reprinted with partially modified from Takeshima and Gyoba [60]).

Digits, composed of line segments, were used as the two targets (Figure 1a). White (43.5 cd/m^2^) digits were presented as first target (T1), and black (1.7 cd/m^2^) digits were used as second target (T2). The color of T1 was different from that of the other visual stimuli to decrease the effort required to distinguish T1 from the distracters. Letters of the alphabet composed of black line segments were presented as distracters (Figure 1b) and line segments from which the digits and letters were made were also used to create the mask stimulus (Figure 1c). The targets, distracters, and mask stimulus were within 1.0×1.0 deg. These visual stimuli and a black fixation cross (about 0.4×0.4 deg) were presented on a gray (17.9 cd/m^2^) background. Visual stimuli were presented 2.5 deg to each side of the fixation. The auditory stimulus was a pure tone that was presented for 50 ms (including ramp time of 5 ms at the start and end of the sound wave envelope), with a frequency of 1250 Hz, and sound pressure level of 75 dB. The onsets of the visual and auditory stimuli were synchronized.

### Procedure

A schematic representation of a trial is shown in Figure 1d. The participants initiated the trials by pressing the “5” key on the computer keyboard. The fixation cross was presented at the center of the screen for a fixed period of 500 ms, immediately followed by the presentation of two RSVP streams on either side of the fixation. The RSVP streams consisted of 10 elements. Each element was presented for 67 ms, followed by a 33 ms blank interval, presentation of the mask for 50 ms, and then another 100 ms blank interval. This resulted in stimulus onset asynchronies of 250 ms between the RSVP elements. Each RSVP stream started with the presentation of a randomly chosen distracter from the letter set (without replacement). Then, the T1 element was presented as either the second, third or fourth element, immediately followed by a variable number of distracters (depending on the lag variable). Finally, the T2 element was presented, followed by the remainder of the distracter elements. Thus, the total number of elements in the RSVP stream was always 10. We set the number of lags (the number of interleaved elements between first and second stimulus) as one, two, and five. Thus, the temporal distances between T1 and T2 were 250, 500, and 1250 ms. Targets were also randomly drawn from the digit set, without replacement. T1 was presented either in the left RSVP stream (for half of the trials), or in the right one. Similarly, the T2 presentations were distributed between the left and right RSVP streams. Thus, T2 was presented in the same visual field as T1 for half the trials. The auditory stimulus was presented simultaneously with the onset of T2. After viewing the RSVP streams, participants were asked to report the digit identities of T1 and T2 by pressing the corresponding keys on the keyboard. Each participant completed 12 trials for each condition, Tone (2; Tone-absent or Tone-on-T2)×T1 visual field (T1 VF: 2; left or right)×T2 visual field (T2 VF: 2; left or right)×Lag (3; lag-1, lag-2, or lag-5) for 288 trials.

### Results

**Figure 2:**
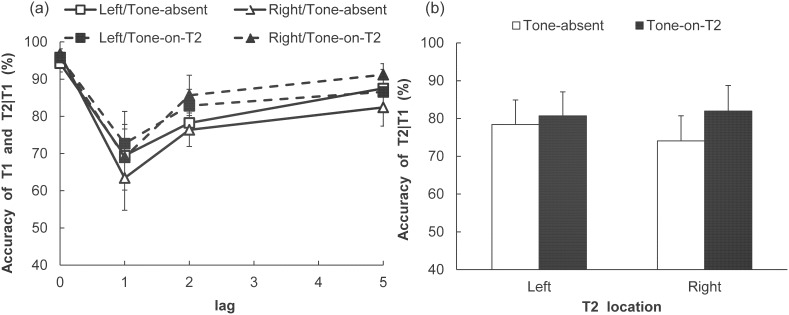
Performance on T1 and T2 identification in Experiment 1A. (a) T1 accuracy and T2 accuracy (given that T1 is correct) in each of the Tone, Visual field, and Lag conditions. (b) The T2 accuracy (given that T1 is correct) in each of the Tone and T2 visual field conditions. Vertical axes indicate T2 accuracy (percent correct). Error bars represent standard errors of the mean (*n* = 9: With the permission from JPS, reprinted with partially modified from Takeshima and Gyoba [60]).

The accuracy in identifying T1 and T2, with the latter contingent upon T1 being correct, was calculated for each of the conditions (Figure 2). The T1 identification performance was high in each condition. A two-way analysis of variance (ANOVA) with Tone (2)×T1 VF (2) indicated no significant main effects (Tone: *F* (1, 8) = 0.13, *p* = .73, η_p_^2^ = .02; T1 VF: *F* (1, 8) = 3.18, *p* = .11, η_p_^2^ = .28) and no significant interaction (*F* (1, 8) = 2.86, *p* = .13, η_p_^2^ = .25).

For T2 identification performance, a three-way ANOVA with Tone (2)×T2 VF (2)×Lag (3) was conducted. The main effects of Tone (*F* (1, 8) = 9.01, *p*<.05, η_p_^2^ = .53) and Lag (*F* (2, 16) = 10.37, *p*<.005, η_p_^2^ = .56) were significant. Multiple comparisons (Ryan’s method) indicated that the rate of correct T2 identification was lower in the lag-1 condition than in the lag-2 and lag-5 conditions (*p*<.01 in all comparisons). The difference in accuracy was marginally significant between lag-2 and lag-5 (*p* = .15). Moreover, the interaction between Tone and T2 VF was significant (*F* (1, 8) = 7.29, *p*<.05, η_p_^2^ = .48). The simple main effect of Tone was significant in the RVF condition (*F* (1, 16) = 15.73, *p*<.005, η_p_^2^ = .50), indicating that correct identification rate was higher in the Tone-on-T2 condition than in the Tone-absent condition when T2 was presented in the RVF. In contrast, the simple main effect of Tone was not significant in the LVF condition (*F* (1, 16) = 1.36, *p* = .26, η_p_^2^ = .08). The remaining main effects and interactions were not significant. However, there was a marginally significant simple main effect of T2 VF in the Tone-absent condition (*F* (1, 16) = 3.20, *p* = .09, η_p_^2^ = .16).

## Experiment 1B

Experiment 1A showed that the presence of an auditory stimulus improved T2 performance in the RVF. Experiment 1B was then conducted to collect supplemental data by manipulating the Tone and Lag conditions.

### Participants

A group of nine right-handed participants (six women and three men), seven of whom had not taken part in Experiment 1A, participated in Experiment 1B. They reported normal or corrected-to-normal vision and audition. Handedness was assessed with the Edinburgh Handedness Inventory, Mean LQ = 91 (*SD* = 13).

### Stimuli

The visual and auditory stimuli were the same as in Experiment 1A.

### Procedure

The procedure was almost the same as for Experiment 1A. However, the auditory stimulus could be presented simultaneously, either with T1 or with T2. The T2 element was presented at lag-1, lag-3, or lag-5 (after T1). Each participant completed 12 trials for each condition, Tone (2)×T1 VF (2)×T2 VF (2)×Lag (3) for 288 trials.

### Results

**Figure 3:**
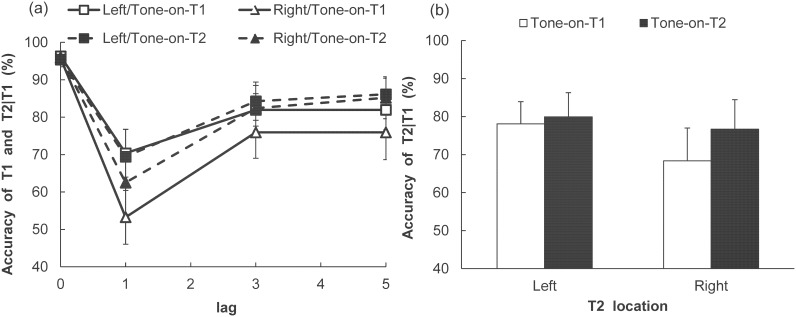
Performance onT1 and T2 identification in Experiment 1B. (a) T1 accuracy and T2 accuracy (given that T1 is correct) in each of the Tone, Visual field, and Lag conditions. (b) T2 accuracy (given that T1 is correct) in each of the Tone and T2 visual field conditions. Vertical axes indicate T2 identification accuracy (percent correct). Error bars represent standard errors of the mean (*n* = 9).

The accuracy in identifying T1 and T2, with the latter contingent upon T1 being correct, was calculated for each of the conditions (results shown in Figure 3). The T1 identification performance was high in each condition. A two-way ANOVA with Tone (2)×T1 VF (2) indicated no significant main effects (Tone: *F* (1, 8) = 0.36, *p* = .57, η_p_^2^ = .04; T1 VF: *F* (1, 8) = 0.11, *p* = .92, η_p_^2^ = .01) nor a significant interaction (*F* (1, 8) = 0.36, *p* = .56, η_p_^2^ = .04).

For T2 identification performance, a three-way ANOVA with Tone (2)×T2 VF (2)×Lag (3) was conducted. The main effects of Tone (*F* (1, 8) = 7.90, *p*<.05, η_p_^2^ = .50), T2 VF (*F* (1, 8) = 6.49, *p*<.05, η_p_^2^ = .45) and Lag (*F* (2, 16) = 13.47, *p*<.001, η_p_^2^ = .63) were significant. Multiple comparisons indicated that the rate of correct T2 identification was lower in the lag-1 condition than in the lag-3 and lag-5 conditions (*p*<.001 for all comparisons). The difference in accuracy was not significant between lag-3 and lag-5 (*p* = .77). Moreover, the interactions between Tone and T2 VF (*F* (1, 8) = 7.06, *p*<.05, η_p_^2^ = .47), and between T2 VF and Lag (*F* (2, 16) = 4.52, *p*<.05, η_p_^2^ = .36), were significant. The simple main effect of Tone was significant in the RVF condition (*F* (1, 16) = 14.56, *p*<.005, η_p_^2^ = .48), indicating that a simultaneous auditory stimulus improved T2 identification performance in the RVF. In contrast, the simple main effect of Tone was not significant in the LVF condition (*F* (1, 16) = 0.72, *p* = .41, η_p_^2^ = .04). Furthermore, the simple main effect of T2 VF was significant in the Tone-on-T1 condition (*F* (1, 16) = 11.88, *p*<.005, η_p_^2^ = .43), indicating that T2 accuracy was higher in the LVF than in the RVF when the auditory stimulus was presented with T1 (i.e., when T2 was presented without an accompanying sound). The simple main effect of T2 VF was also significant in the lag-1 condition (*F* (1, 24) = 14.64, *p*<.001, η_p_^2^ = .38), indicating that accuracy in the RVF was lower than that in the LVF in the lag-1 condition.

### Discussion

In Experiment 1A, we compared the effects of an accompanying tone on T2 identification performance for LVF and RVF presentations in a dual-stream RSVP task. The T2 identification performance was lower at lag-1 than at lag-2 and lag-5. Furthermore, presentation of an auditory stimulus with T2 was found to improve performance in the RVF. In contrast, no effect of the auditory stimulus was observed in the LVF. Therefore, a simultaneous auditory stimulus may facilitate LH visual processing in a dual-stream RSVP task.

Experiment 1B replicated most of the results from Experiment 1A. The facilitating effect of the auditory stimulus was observed in the RVF in both Experiments (1A and 1B). However, in Experiment 1B, T2 identification performance was not improved by the presentation of a sound with T1. Olivers and Van der Burg [15] showed that the presentation of a tone prior to the target did not enhance T2 identification. Therefore, this facilitation effect is not induced by the simple arousal or alerting that may accompany the presentation of any tone. According to the results Experiment 1B, visual field asymmetry in the auditory facilitation effect is a robust phenomenon.

In both Experiments 1A and 1B, the auditory facilitation effect did not depend on the temporal location between T1 and T2. These results confirmed our prediction. Synchronous sound produces robust and salient T2 representation. According to bottleneck model [36], [37], T2 representation is declined and interrupted by distracters in small lag condition. On the other hand, in large lag condition, T2 representation is not affected by distracters. In the present study, T2 accuracy was improved by simultaneous sound in both cases. Therefore, synchronous sound does not only decrease the interruption due to distracters for T2 representation, but also increases the saliency of T2 representation.

In Experiment 1B, T2 accuracy was higher in the LVF than in the RVF when sound was presented with T1. This result replicated the previous studies [38]–[41]. Hollander *et al.* [38] concluded that the AB deficit is related to more spatial processing (for which the LVF has an advantage) than to temporal processing. This LVF advantage for T2 accuracy has been confirmed in an ERP study [42]. In Experiment 1A, the difference between T2 accuracy in the LVF and the RVF was only marginally significant when sound was not presented. However, we did find that the percentage of correct T2 identification was higher in the LVF than the RVF.

Overall the LVF performance was high, which may have been due to superior RH processing in this paradigm. Therefore, the impact of the sound may not have been observed in the LVF because of a ceiling effect. We investigated this possibility in Experiment 2.

## Experiment 2

The results of Experiments 1A and 1B indicated that an auditory stimulus only improved the T2 identification performance in the RVF. However, RH processing is superior to LH processing in dual-stream RSVP tasks [38]–[41]. Therefore, in Experiment 2, we attempted to eliminate a possible ceiling effect in LVF performance by using the paradigm employed by Visser [44]. In this paradigm, T2 performance is lower than in the typical paradigm due to a higher load at T1 identification.

### Participants

A group of nine, right-handed participants (five women and four men), three of whom had not taken part in Experiments 1A and 1B, participated in Experiment 2. They reported normal or corrected-to-normal vision and audition. Handedness was assessed with the Edinburgh Handedness Inventory, Mean LQ = 88 (*SD* = 14).

### Stimuli

**Figure 4:**
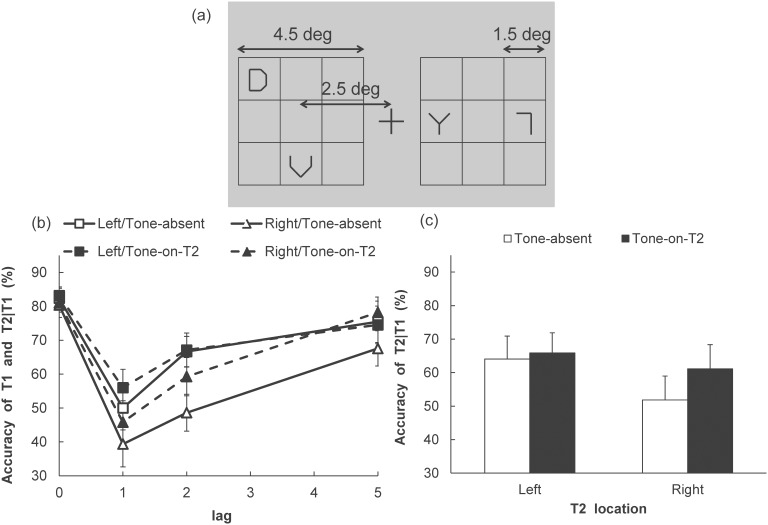
Illustration of T1 display and T1 and T2 identification performance in Experiment 2. (a) T1 display: T1 and three distracters were presented at random locations on a 3×3 virtual matrix. Two visual stimuli were displayed on each side (left or right) of the fixation. (b) T1 accuracy and T2 accuracy (given that T1 is correct) in each of the Tone, Visual field, and Lag conditions. (c) T2 accuracy (given that T1 is correct) in each of the Tone and T2 visual field conditions. The vertical axes indicate the T2 accuracy (percent correct). Error bars represent standard errors of the mean (*n* = 9).

The visual and auditory stimuli were the same as in Experiment 1A. However, the color of T1 was black in the present experiment, and the target was presented simultaneously with three distracters (see Figure 4a). The locations of T1 and the distracters were determined randomly using a 3×3 (4.5×4.5 deg) virtual matrix.

### Procedure

The trial sequence was almost the same as in Experiment 1A. However, a blank display was presented (instead of the mask stimulus) for 183 ms immediately after T1. Visser [44] proposed that competition between T1 and the distracter induces a delay of T1 processing, leading to a decline in T2 performance. However, T1 masking decreases this competition. Therefore, in the current experiment, the mask after T1 was replaced with a blank display. Each participant completed 12 trials for each of the conditions, Tone (2)×T1 VF (2)×T2 VF (2)×Lag (3), for 288 trials.

### Results

The accuracy in identifying T1 and T2, with the latter contingent upon T1 being correct, was calculated for each of the conditions (results shown in Figures 4b and 4c). The T1 identification performance was high in each condition. A two-way ANOVA, with Tone (2)×T1 VF (2), indicated that no significant main effects (Tone: *F* (1, 8) = 0.27, *p* = .62, η_p_^2^ = .03; T1 VF: *F* (1, 8) = 1.02, *p* = .34, η_p_^2^ = .11) and no significant interaction (*F* (1, 8) = 0.03, *p* = .96, η_p_^2^ = .01). For a manipulation check, we compared T1 identification performance in Experiment 1A with that of Experiment 2 using a three-way ANOVA, Experiment (2) ×Tone (2)×T2 VF (2). The main effect of Experiment was significant (*F* (1, 16) = 17.13, *p*<.001, η_p_^2^ = .52), indicating that T1 identification performance was lower in Experiment 2 than in Experiment 1A. Therefore, the experimental manipulation (greater load at T1 identification) was relevant. However, other main effects and interactions were not significant.

For the T2 identification performance, a three-way ANOVA with Tone (2)×T2 VF (2)×Lag (3) was conducted. The main effects of Tone (*F* (1, 8) = 5.92, *p*<.05, η_p_^2^ = .42), T2 VF (*F* (1, 8) = 13.62, *p*<.01, η_p_^2^ = .63), and Lag (*F* (2, 16) = 17.03, *p*<.001, η_p_^2^ = .68) were significant. Multiple comparisons indicated that the accuracy was higher with increased lag between T1 and T2 (*p*<.05 for all comparisons). Moreover, the interactions between Tone and T2 VF (*F* (1, 8) = 6.36, *p*<.05, η_p_^2^ = .44), and between T2 VF and Lag (*F* (2, 16) = 4.03, *p*<.05, η_p_^2^ = .33) were significant. The simple main effect of Tone was significant under the RVF condition (*F* (1, 16) = 11.64, *p*<.005, η_p_^2^ = .42), indicating that the auditory stimulus improved T2 identification performance in the RVF. In contrast, the simple main effect of Tone was not significant in the LVF condition (*F* (1, 16) = 0.47, *p* = .50, η_p_^2^ = .03). Furthermore, the simple main effect of T2 VF was significant in the Tone-absent condition (*F* (1, 16) = 19.96, *p*<.001, η_p_^2^ = .56), indicating that T2 accuracy was higher in the LVF than in the RVF when the auditory stimulus was not presented. The simple main effect of T2 VF was also significant in lag-1 (*F* (1, 24) = 10.2, *p*<.005, η_p_^2^ = .30) and lag-2 (*F* (1, 24) = 15.79, *p*<.001, η_p_^2^ = .40) conditions, indicating that accuracy of the RVF was lower than that of the LVF in lag-1 and lag-2 conditions.

### Discussion

In Experiment 2, T2 identification was made more difficult by the high load at T1 identification. The overall performance on T2 identification was lower in the present experiment, as compared to Experiments 1A and 1B. Nevertheless, the auditory facilitation effect was observed only in the RVF. Therefore, we propose that this effect of an auditory stimulus occurs uniquely in the RVF, and that the absence of auditory facilitation in the LVF is not due to a ceiling effect. In this experiment, we observed consistently poor performance in the RVF when compared to the LVF.

## Experiment 3

In Experiments 1A, 1B, and 2, improvement in the T2 identification performance due to presentation of an auditory stimulus was observed only in the RVF. This effect may have been induced by an auditory facilitation effect on inferior processing in the LH. However, stronger audio-visual interaction has been reported over left parieto-occipital cortex when compared with the right [45]. Moreover, visual field asymmetry of the auditory facilitation effect may be related to the processing specializations of each of the cerebral hemispheres, (e.g., because the LH is specialized for temporal processing) [30], [31]. Thus, in Experiment 3, we investigated visual field asymmetry of the auditory facilitation effect using a spatial localization task. In this case, if the auditory facilitation effect was dependent on hemispheric specialization, then T2 performance improvement should be observed only in the LVF; unlike the LH, the RH is dominant in spatial processing [25]–[27]. However, if improved T2 performance was observed only in the RVF, then the facilitation effect could be attributed to other influences.

### Participants

A group of eight, right-handed participants (four women and four men), six of whom had not taken part in Experiments 1A, 1B, or 2, participated in Experiment 3. They reported normal or corrected-to-normal vision and audition. Handedness was assessed with the Edinburgh Handedness Inventory, Mean LQ = 87 (*SD* = 11).

### Stimuli

The visual and auditory stimuli were the same as in Experiment 1A.

### Procedure

**Figure 5:**
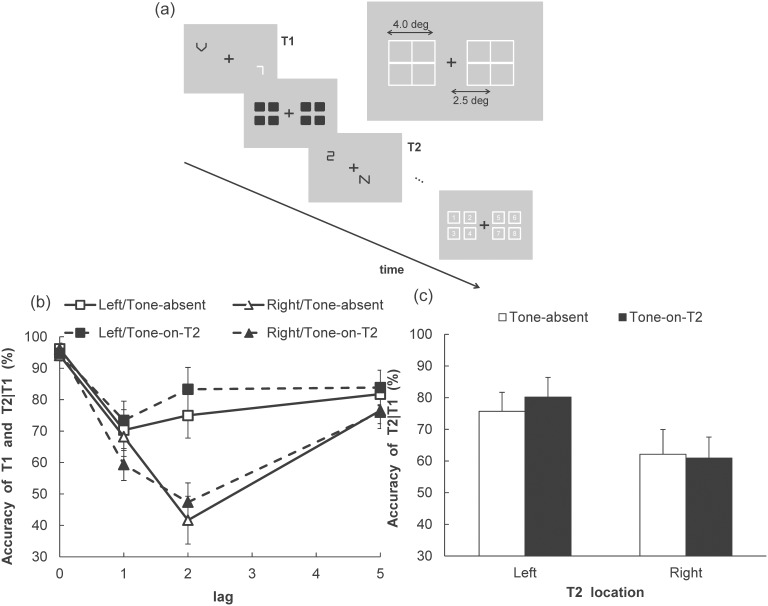
Outline of the paradigm and T1 and T2 localization performance in Experiment 3. (a) Trial sequence: Participants were presented with two RSVP streams and were instructed to determine the location of the two digit targets among a series of letters. The visual stimuli were presented randomly at one of the four locations on both the left and right sides on the display. T2 was presented at lag-1, lag-2, or lag-5, after T1. A tone was presented simultaneously with the onset of T2. (b) T1 accuracy and T2 accuracy (given that T1 is correct) in each of the Tone, Visual field, and Lag conditions. (c) T2 accuracy (given that T1 is correct) in each of the Tone and T2 visual field conditions. Vertical axes indicate the T2 accuracy (percent correct). Error bars represent standard errors of the mean (*n* = 8: With the permission from JPS, reprinted with partially modified from Takeshima and Gyoba [60]).

The procedure was almost the same as in Experiment 1A. However, the visual stimuli were presented randomly, within a 2×2 (4.0×4.0 deg) virtual matrix (see Figure 5a). The participants’ task was to report the locations in which T1 and T2 were presented. Numbers from one to eight were assigned to the locations of the visual stimuli (Figure 5a) and participants pressed the corresponding key to report the locations of T1 and T2. Each participant completed 12 trials for each of the conditions, Tone (2)×T1 VF (2)×T2 VF (2)×Lag (3), for 288 trials.

### Results

The accuracy in localizing T1 and T2, with the latter contingent upon T1 being correct, was calculated for each of the conditions (results shown in Figures 5b and 5c). The T2 localization performance was high in each condition. A two-way ANOVA with Tone (2)×T1 VF (2) indicated no significant main effects (Tone: *F* (1, 7) = 0.13, *p* = .73, η_p_^2^ = .02; T1 VF: *F* (1, 7) = 0.28, *p* = .62, η_p_^2^ = .04) and no a significant interaction (*F* (1, 7) = 1.61, *p* = .24, η_p_^2^ = .19).

For the T2 localization performance, a three-way ANOVA with Tone (2)×T2 VF (2)×Lag (3) was conducted. The main effects of T2 VF (*F* (1, 7) = 11.72, *p*<.05, η_p_^2^ = .63) and Lag (*F* (2, 14) = 15.67, *p*<.001, η_p_^2^ = .69) were significant. Furthermore, the interactions between Tone and T2 VF (*F* (1, 7) = 5.71, *p*<.05, η_p_^2^ = .45), and between T2 VF and Lag (*F* (2, 14) = 13.24, *p*<.001, η_p_^2^ = .65), were significant. The simple main effect of Tone was significant in the LVF condition (*F* (1, 14) = 8.08, *p*<.05, η_p_^2^ = .37), indicating that T2 localization performance in the LVF was higher when the tone was presented than when the tone was absent. In contrast, the simple main effect of Tone was not significant in the RVF condition (*F* (1, 14) = 0.59, *p*<.46, η_p_^2^ = .04). Furthermore, the simple main effects of T2 VF were significant in both Tone conditions (Tone-absent: *F* (1, 14) = 7.51, *p*<.05, η_p_^2^ = .35; Tone-in-T2: *F* (1, 14) = 15.22, *p*<.005, η_p_^2^ = .52), indicating that, in both the Tone conditions, accuracy in T2 localization was higher when T2 was presented in the LVF than in the RVF in both the Tone conditions. The simple main effect of Lag was also significant in the RVF condition (*F* (2, 28) = 25.89, *p*<.001, η_p_^2^ = .65). Multiple comparisons revealed that T2 localization performance was lower at lag-2 than at lag-1 and lag-5 (*p*<.001 for all comparisons. Performance at lag-5 was higher than at lag-1 (*p*<.01) in the RVF. Additionally, the simple main effect of T2 VF was significant in the lag-2 condition (*F* (1, 21) = 33.76, *p*<.001, η_p_^2^ = .62), indicating that accuracy was higher in the LVF than in the RVF.

### Discussion

Unlike in Experiments 1A, 1B, and 2, an auditory facilitation effect was observed in the LVF in the present experiment. If the improved T2 performance in the previous experiments was attributed to magnitude of the left lateralized audio-visual interaction [45], then the facilitation effect observed in the RVF alone. Therefore, we propose that the auditory facilitation effect is related to hemispheric asymmetry in processing specialization. The task in Experiments 1A, 1B and 2 was to identify digits in the RSVP stream, requiring both linguistic and temporal processing. In contrast, the task in Experiment 3 was to localize the position of the presented digits, thus requiring spatial processing. In the present experiment, linguistic and temporal processing was required to distinguish the target from distracters in the RSVP stream. However, accurate identification of the target was not necessary for the localization task, making spatial processing the relatively dominant form of processing. Previous research has shown that a RVF-LH advantage for letter identification is reversed when visuo-spatial demand increases [46], [47]. Therefore, the LVF-RH activation, which is dominant for spatial processing [25]–[27], was likely superior to the RVF-LH activation in the current experiment. Thus, the auditory facilitation effect occurred in the dominant side according to the required processing type (i.e., the LVF where there is specialization for spatial processing). In addition, we propose another possibility that the sound modulated visual processing differently between the LVF and RVF. In target localization task, target was initially detected and specified in the RSVP stream, and then was localized. According to the hemispheric specialization, attention tends to direct for linguistic and temporal detection process in the RVF. On the other hand, in the LVF, attention tends to direct for localization process performing after detecting and specifying process. Therefore, the auditory facilitation effect would be observed only at the RVF in target identification task and only at the LVF in target localization task, respectively.

## General Discussion

The present study examined the relationship between the facilitating effect of audio-visual integration and hemispheric asymmetry in attentional processes. The results of Experiments 1A, 1B, and 2 indicated that T2 identification performance was improved by an auditory stimulus presented in the RVF alone. In addition, this improvement was not attributed to increased arousal or an alerting effect of the tone [15]. Furthermore, the observed visual field asymmetry was not the result of a ceiling effect due to an RH processing advantage in the dual-stream RSVP tasks. In contrast, the results of Experiment 3 showed a facilitating effect of a simultaneous tone in the LVF.

For the RVF, T2 identification performance was facilitated by sound. In contrast, simultaneous sound improved T2 localization performance in the LVF. Previous research has shown that, in the dual-stream RSVP task, performance is poor in the RVF when compared with the LVF [38]–[41]. A salient sound captures the onset of the simultaneously presented visual item and thus segregates it from the RSVP stream [18]. The RVF-LH plays a dominant role in temporal processing [30], [31]. Further, the results of Experiments 1 and 2 may be explained by the compensation of an auditory stimulus for poor temporal processing in the dual-stream RSVP task. However, this interpretation does not apply for the results of Experiment 3, in which the performance of the LVF was improved by sound.

In addition to temporal processing, the RVF-LH plays a dominant role in linguistic processing. In contrast, the LVF-RH dominates spatial processing [24]–[27]. In Experiments 1 and 2, the task was to identify the two target digits in the RSVP stream; thus it involved linguistic and temporal processing. In Experiment 3, participants were asked to localize the two target digits but the task also involved linguistic and temporal processing. However, research has shown that hemispheric advantage for linguistic processing is reversed by increasing visuo-spatial demand [46], [47], and therefore, there was a relative requirement for spatial processing in Experiment 3. In our study, there was a correspondence between hemispheric specializations and visual field asymmetry observed in the auditory facilitation effect. We propose that a salient stimulus (i.e., the auditory stimulus) facilitates processing in the hemisphere that is dominant for that perceptual processing, as evidenced by visual field asymmetry in the auditory facilitation effect.

Hemispheric specialization has also been reported to occur in the context of both global and local processing [48]–[50]. According to these studies, global perception is dominant in the LVF-RH, while local perception is dominant in the RVF-LH. The T2 localization depends more on global processing than does T2 identification, because the spatial range where the target is presented is broader in the former task. The visual field asymmetry observed in our study also corresponds to the hemispheric specialization in this respect. The results of our study can be interpreted according to this visual field asymmetry for processing specialization.

According to visual processing specialization, visual field asymmetry in the auditory facilitation effect would be induced by cross-modal attentional spread [35]. In cross-modal attentional spread, task-irrelevant sound is grouped with synchronous attended visual object, even if the auditory stimulus is presented from a different location. Attention toward the visual object spreads the auditory stimulus and increases activation at auditory cortices multisensory processing is then enhanced. In the present study, hemispheric specialization for sensory processing would provide the cue to attend to one or the other visual hemi-field. Therefore, the RVF, which dominates during temporal and linguistic processing, is attended to in the target identification task. However, in the target localization task, attention is directed toward the LVF, which advantages spatial processing. Because this later attentional selection depends on hemispheric specialization, it would induce visual field asymmetry in the auditory facilitation effect by cross-modal attentional spread.

Feedback projections from primary auditory cortex (A1) to V1 are related to audio-visual interactions [51]–[53]. For example, V1 is activated when an illusory flash is induced by an auditory stimulus [54], [55]. In addition, a synchronous auditory stimulus facilitates visual object representation [3] and improves visual performance in the early stages of processing [33], [34]. The reduction in T2 performance levels in RSVP tasks is caused by impairment in the ability to form a T2 representation while T1 is being processed [36], [37]. Thus, feedback from the auditory cortex to the visual cortex could also be related to the improvement in T2 performance by increasing the magnitude of T2 representation. In addition to later attentional selection by cross-modal attentional spread, this early effect of audio-visual integration would be an important contributor to the auditory facilitation effect.

Auditory stimuli strongly influence visual perception when two sensory signals occur in the same location [4]. Neural activity also increases the spatial correspondence between two sensory inputs [56]–[58]. In our study, the visual targets were presented in the left or the right hemi-field. However, the auditory stimulus was conveyed binaurally through headphones. In this paradigm, the spatial correspondence between visual and auditory sensations was poor. Therefore, the facilitating effect of the audio-visual integration that we observed might be weaker than in conditions where the visual and auditory stimuli are presented from the same location.

In Experiment 3, accuracy in the RVF decreased most substantially at lag-2. This result may reflect the visual field asymmetry in the attentional mechanism. Dell’Acqua *et al*. [59] have shown that allocation of attention is suppressed during T1 processing in the AB paradigm. However, in their study, the difference between left and right visual field was not examined. Thus, there may have been a time-related visual field asymmetry in the suppression for spatial attention. Further research is required to elucidate the hemispheric asymmetry of attentional mechanisms in the spatial domain.

Audio-visual integration is fundamental to stable and efficient perception of the outer environment. Many previous studies have indicated that auditory information compensates for the low reliability of visual stimuli [9]–[11]. However, in the present study, an auditory stimulus influenced the visual performance when T2 was presented at the visual hemi-field which had processing specialization for the task. Thus, we observed that the auditory stimulus facilitated visual processing in two ways: (1) it compensated for the ambiguity of visual information and (2) it reinforced the dominant function of visual processing. Based on this behavioral data, future work should confirm the role of hemispheric specialization on attentional capture using ERPs because this method provides superior temporal resolution compared with other brain imaging methods such as fMRI.
